# An Attention-Based Bidirectional Feature Fusion Algorithm for Insulator Detection

**DOI:** 10.3390/s26020584

**Published:** 2026-01-15

**Authors:** Binghao Gao, Jinyu Guo, Yongyue Wang, Dong Li, Xiaoqiang Jia

**Affiliations:** 1 College of Information Engineering, Inner Mongolia University of Technology, Hohhot 010080, China; 202310203036@imut.edu.cn (B.G.); 202310203038@imut.edu.cn (J.G.); 202310203044@imut.edu.cn (Y.W.); jiaxiaoqiang@imut.edu.cn (X.J.); 2 Inner Mongolia Key Laboratory of Intelligent Perception and System Engineering, Hohhot 010080, China

**Keywords:** insulator detection, deep learning, multi-scale features, feature fusion, attention mechanism

## Abstract

To maintain reliability, safety, and sustainability in power transmission, insulator defect detection has become a critical task in power line inspection. Due to the complex backgrounds and small defect sizes encountered in insulator defect images, issues such as false detections and missed detections often occur. The existing You Only Look Once (YOLO) object detection algorithm is currently the mainstream method for image-based insulator defect detection in power lines. However, existing models suffer from low detection accuracy. To address this issue, this paper presents an improved YOLOv5-based MC-YOLO insulator detection algorithm. To effectively extract multi-scale information and enhance the model’s ability to represent feature information, a multi-scale attention convolutional fusion (MACF) module incorporating an attention mechanism is proposed. This module utilises parallel convolutions with different kernel sizes to effectively extract features at various scales and highlights the feature representation of key targets through the attention mechanism, thereby improving the detection accuracy. Additionally, a cross-context feature fusion module (CCFM) is designed, where shallow features gain partial deep semantic supplementation and deep features absorb shallow spatial information, achieving bidirectional information flow. Furthermore, the Spatial-Channel Dual Attention Module (SCDAM) is introduced into CCFM. By incorporating a dynamic attention-guided bidirectional cross-fusion mechanism, it effectively resolves the feature deviation between shallow details and deep semantics during multi-scale feature fusion. The experimental results show that the MC-YOLO algorithm achieves an mAP@0.5 of 67.4% on the dataset used in this study, which is a 4.1% improvement over the original YOLOv5. Although the FPS is slightly reduced compared to the original model, it remains practical and capable of rapidly and accurately detecting insulator defects.

## 1. Introduction

**Figure 1:**
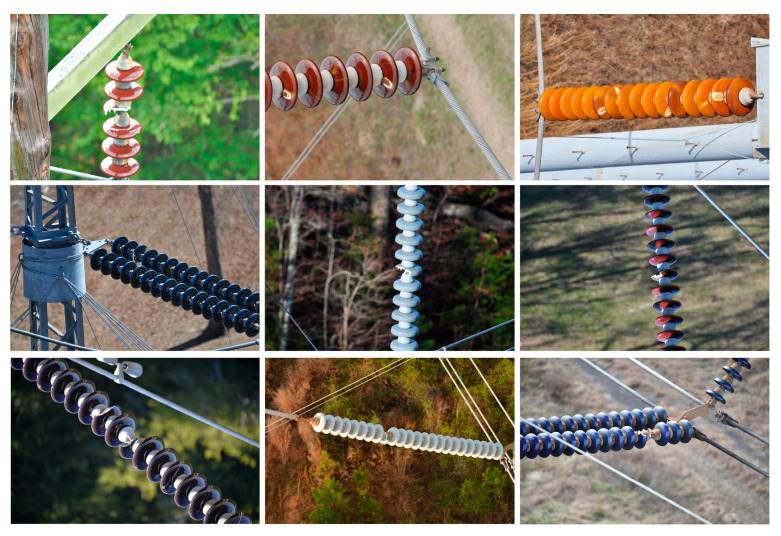
Typical Defect Images of Insulators.

With the rapid development of high-speed rail technology in China, the area in which contact network power transmission lines are installed has been expanding, making it especially important to ensure the safe and stable operation of the contact network. As a critical component of the overhead contact system, insulators provide support for conductors and facilitate electrical insulation within power transmission circuits. However, prolonged exposure to harsh external environments can cause insulators to become susceptible to damage, flashover, and other defects (see Figure 1). These issues compromise the insulation performance, posing significant threats to the safety and reliability of transmission lines. Consequently, regularly conducting inspections is essential in the expeditious identification of insulator defects and the implementation of appropriate measures. Conventional manual field inspections are characterised by inefficiency and an inability to adequately address the demands of insulator defect detection. The integration of machine learning and artificial intelligence technologies has effectively reduced the complexity of insulator inspection tasks, promoted personnel safety, and significantly enhanced inspection efficiency [1,2,3,4].

In recent years, deep learning algorithms have achieved significant advances in image recognition and object detection. Object detection methods based on deep learning offer advantages such as high detection accuracy and strong generalisation capabilities, leading to their widespread application in insulator defect detection [5]. The starting point for the development of these algorithms was two-stage detectors, including the Fast Region-based Convolutional Neural Network (Fast R-CNN) proposed by Ross et al. [6], the Faster R-CNN proposed by Ren et al. [7], and the Cascade R-CNN proposed by Cai et al. [8]. The functioning of these algorithms is initiated by the selection of candidate regions prior to the performance of object localisation and classification. The protracted nature of the candidate region selection process gives rise to a comparatively elevated computational complexity. Subsequent single-stage detectors concurrently perform object bounding box localisation and recognition tasks, offering faster detection speeds and greater simplicity. Notable examples include YOLOv1 by Redmon et al. [9], YOLOX by Ge et al. [10], PP-YOLO by Long et al. [11], YOLOv7 by Wang et al. [12], and SSD by Liu et al. [13].

At present, research in this area is focused in two primary directions. Firstly, there is an emphasis on enhancing detection accuracy. Secondly, there is a focus on the development of lightweight models. Hu et al. [14] proposed a methodology combining Faster R-CNN and U-shaped Network. The former is utilised for the location of glass insulator strings, whilst the latter performs precise pixel classification on cropped images of differing scales. This approach has been demonstrated to outperform certain classical algorithms in terms of insulator localisation and crack location determination. Guo et al. [15] addressed the challenges of indistinct target features and low detection accuracy for small objects in drone inspections by proposing an improved YOLOv5-based insulator defect detection algorithm. This approach incorporates ConvNeXt architecture into the backbone network with a view to enhancing feature extraction capabilities and integrates a coordinate attention mechanism to boost small object detection performance. Wang et al. [16] proposed a methodology for the detection of glass insulator defects, which is based on a dynamic difference algorithm. The apparatus utilises a rotating control platform and an inverted dam-type LED light source to suppress complex backgrounds, combined with a support vector machine classifier for defect identification. The experimental results demonstrate that this method achieves a detection accuracy of 2 mm and a processing speed of 10 samples per minute, effectively meeting industrial automation inspection requirements. The intricacy of glass insulator shapes and the heterogeneity of defects present considerable challenges in automated detection. Test results confirm that this detection method achieves 2 mm accuracy and 10 samples per minute, enabling the effective detection and identification of glass insulator defects and satisfying industrial automation production needs.

In the area of lightweight and real-time detection, Ma et al. [17] proposed a lightweight YOLOv4 detection model to address the issues of large backbone architecture and high parameter count in YOLOv4. This approach incorporates GhostNet as the feature extraction network, thereby significantly reducing the parameter count and accelerating inference while maintaining detection accuracy. Furthermore, the K-means++ clustering algorithm is employed to optimise anchor box sizes, and Quality Focal Loss is integrated into the loss function to enhance model performance. Jia et al. [18] proposed the lightweight detection model MDD-YOLOv3, in which standard convolutions in the YOLOv3 backbone were replaced with deep separable convolutions to construct the D-Darknet53 backbone. The experimental results demonstrated that the model under investigation achieved a slight improvement in detection accuracy, whilst simultaneously leading to a significant increase in detection speed. These findings demonstrate the efficacy of the proposed method in rapidly and accurately identifying and locating insulators against complex backgrounds. As demonstrated in the preceding discussion, the aforementioned methods have achieved varying degrees of improvement in insulator and defect detection performance. However, it is important to note that these methods primarily address a limited range of defect types. Furthermore, inspection images have characteristics such as diverse defect types and small defect scales. Existing algorithms have proven ineffective in the context of extracting features for multiple defect targets, a situation which gives rise to the possibility of issues such as the failure to detect insulator defects or the erroneous detection of such defects. Moreover, contemporary models are characterised by elevated computational complexity.

The efficacy of these algorithms in enhancing the detection of insulators has been demonstrated to a certain extent. However, their utilisation of image features remains somewhat inadequate, particularly in terms of leveraging global contextual information within images. The following modules are proposed in this paper to enhance the baseline YOLOv5 model:

- An effective multi-scale attention-based convolutional fusion module is proposed. It employs a parallel multi-scale convolutional branch structure to extract features at different scales. Through an attention mechanism, it adaptively assigns weights to features across scales, performs weighted summation across the entire branch, and applies residual projection. This approach enhances more meaningful features while suppressing irrelevant ones, thereby improving feature effectiveness.
- A novel cross-context feature fusion module is proposed, designed to guide and adaptively adjust contextual information during multi-scale feature fusion. Through the SCDAM attention mechanism, the module captures and leverages crucial contextual information during feature fusion, thereby enhancing the effectiveness of feature representations. This effectively guides the model to learn information about detection targets, improving detection accuracy. Simultaneously, through weighted feature re-organisation operations, the module enhances the discriminative capability of feature maps.

## 2. YOLOv5 Architecture

**Figure 2:**
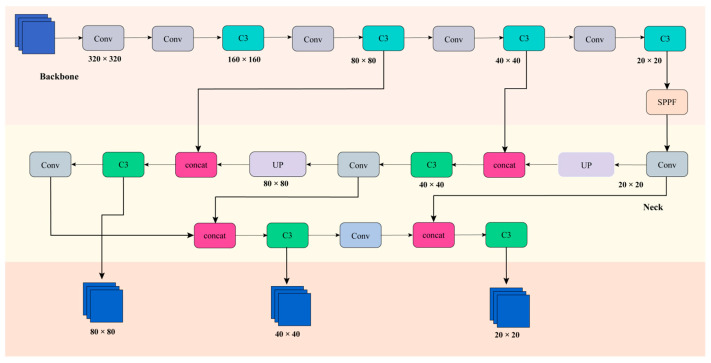
YOLOv5 network structure diagram.

YOLOv5 is a single-stage object detection algorithm characterised by its compact model size, fast processing speed, and high accuracy. As illustrated in Figure 2, the architecture employs a backbone network, which is primarily utilised for feature extraction, resulting in the generation of feature maps that include a variety of semantic information. The Neck component serves to fuse multi-scale features, thereby constructing a feature pyramid. The YOLOv5 Neck architecture employs the FPN + PAN structure. The employment of a Feature Pyramid Network (FPN) to extract high-level semantic feature maps is complemented by the utilisation of a Path Aggregation Network (PAN) to supplement low-level object information. This approach serves to enhance and reinforce the capabilities of localisation. The Head (output stage) serves as the final prediction component, determining the category and location of objects of varying sizes based on feature maps of different dimensions.

## 3. MC-YOLO Model Architecture

**Figure 3:**
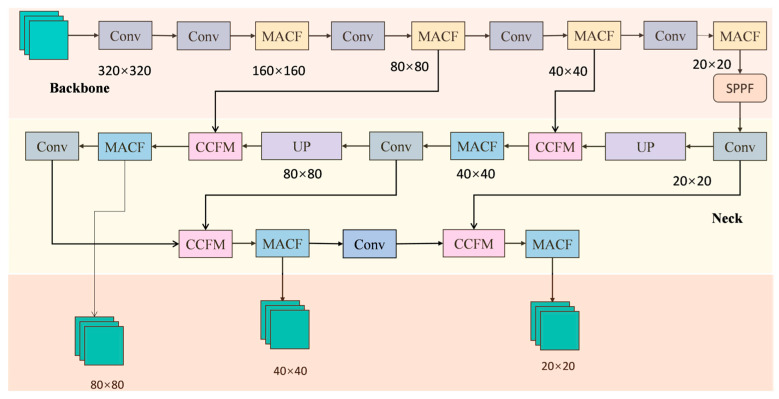
MC-YOLO network structure diagram.

The selection of YOLOv5s as the base model for refinement was informed by its high accuracy and real-time performance, leading to the development of the enhanced MC-YOLO model. During the feature fusion process, the YOLOv5 algorithm necessitates continuous feature map downsampling; however, a solitary sampling method inevitably results in feature loss. In order to optimise the number of features, MC-YOLO introduces a multi-scale attention convolution fusion module, providing the network with a rich source of information. Concurrently, a cross-context feature fusion approach is introduced to mitigate feature loss during feature concatenation in the original YOLOv5 model, thereby endowing the model with more advanced and comprehensive feature fusion capabilities. The block diagram of the MC-YOLO algorithm is shown in Figure 3. In the feature extraction component, the original C3 feature extraction module is replaced with the multi-scale attention convolutional fusion module (MACF) to enhance the model’s feature extraction capability. The CCFM has been demonstrated to capture and utilise crucial contextual information during feature fusion, thereby enhancing the effectiveness of feature representations. SCDAM has been proposed as a means of amplifying important features and enhancing the discriminative power of feature maps.

### 3.1. Multi-Scale Attention Convolution Fusion (MACF) Module

**Figure 4:**
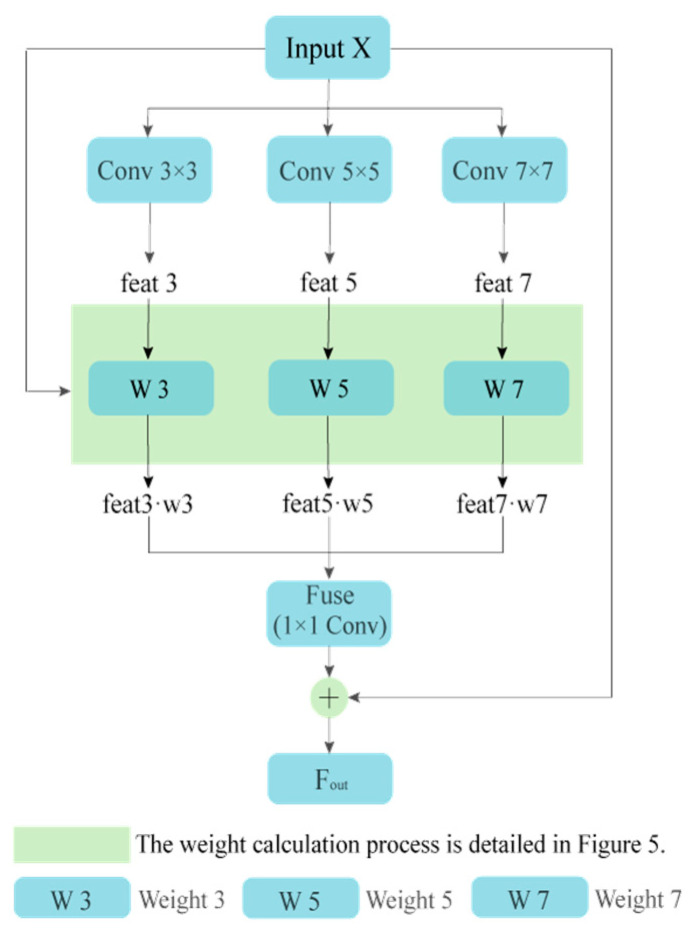
Diagram of the MACF module.

The feature extraction module is responsible for the extraction of feature information from input images, and the subsequent conversion of image data into high-level representations that are rich in semantic information, thus facilitating the efficient execution of subsequent detection tasks. Compared to single-branch structures, multi-branch architectures have been demonstrated to be more effective in capturing features. This paper presents a multi-scale attention convolution fusion (MACF) module to enhance the model’s representational capability for objects at different scales while maintaining lightweight and portable properties. This module employs a parallel multi-scale convolutional branch structure, utilising an attention mechanism to adaptively assign weights to features at different scales. Residual connections are introduced at the output to ensure gradient stability. Following the incorporation of feature map input, convolutions with distinct kernel sizes are employed to facilitate the extraction of features across three parallel branches. It is imperative to note that each individual branch undergoes a process of feature weighting with the objective of retaining effective information. Convolutional operations subsequently fuse the output feature maps into a unified output feature. The model architecture is illustrated in Figure 4.

The input feature X is passed through three parallel convolutional branches with kernel sizes of 3 × 3, 5 × 5, and 7 × 7, respectively, generating multi-scale features feat3, feat5, and feat7. The attention weight generation module performs joint modelling of these multi-scale features to obtain the corresponding weight coefficients w1, w2, and w3, and adaptively fuses the features at each scale through weighted summation to produce the fused feature Fuse. Finally, the fused feature is combined with the input via a residual connection and passed through an activation function to output the final feature Fout. This Fout serves as the output of the MACF module in the overall architecture (Figure 3). Concurrently, it guarantees that the configuration of input and output feature maps remains constant, thereby minimising feature loss in images of small objects. This contributes to enhanced model accuracy in object detection tasks, particularly in complex scenes.

Subsequent to multi-scale feature extraction, the attention mechanism assigns a weighting to features from each branch, thereby facilitating feature selection and enhancement. This process serves to amplify more meaningful features and thereby improve the effectiveness of feature representation.

**Figure 5:**
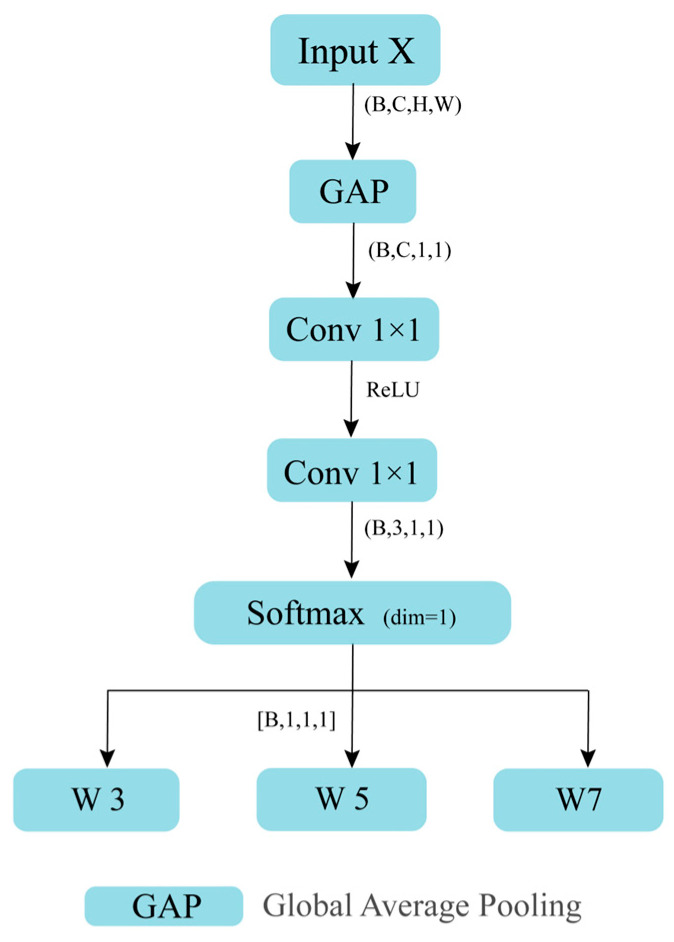
Diagram of how weights are obtained.

As demonstrated in Figure 5, the generation of attention weights within the Attention block is contingent on the global information of the input feature x. The configuration of the input feature $x\in R^{B\times C\times H\times W}$ is represented by $\left[B,C,H,W\right]$, with C denoting the number of channels. The initial procedure involves the implementation of Global Average Pooling (GAP) on x, with the objective of acquiring the feature representation $\left[B,C,1,1\right]$. This process entails the compression of information within the spatial dimension, whilst ensuring the preservation of the global response of each individual channel. The following Formula (1) illustrates this process. S represents the average activation of each channel. Subsequently, a bottleneck structure composed of two 1 × 1 convolutional layers and ReLU activation functions maps the channel features from C dimensions to c_ dimensions and then further maps them to three dimensions to represent the weight scores of the three convolutional branches (3×3,5×5,7×7). SoftMax (dim = 1) performs normalisation along the channel dimension, thereby enabling the three weights to compete numerically while satisfying the constraint that their sum equals 1, as demonstrated in Formula (2).

(1) $$s_{b,c}=\frac{1}{HW}\sum_{i=1}^{H}\sum_{j=1}^{W}x_{b,c,i,j}$$

(2) $$\sum_{k=1}^{3}w_{b,k}=1\;w_{b,k}>0\;$$

This process consequently yields the attention weights $\left[w3,\;w5,\;w7\right]$, as illustrated in Equation (3), where GAP(⋅) denotes the global average pooling operation. The attention weights are multiplied with their corresponding features (feat3, feat5, and feat7) to obtain the final multi-scale feature fusion (see Equation (4)). This approach facilitates the integration of different receptive field branches through adaptive weighted fusion, thereby enabling the model to dynamically adjust the contribution of multi-scale information based on input features.

(3) [w3,w5,w7]=Softmax(Conv(ReLU(Conv(GAP(x)))))

(4) $$f_{fuse}=w3\cdot feat3+w5\cdot feat5+w7\cdot feat7$$

### 3.2. Cross-Context Fusion Module (CCFM)

In the context of object detection tasks, the accurate identification of targets of varying scales frequently necessitates the integration of feature maps from disparate levels to concurrently capture high-level semantic information and low-level spatial details. However, during the process of feature extraction, as convolutional neural networks become more complex, the spatial resolution of feature maps undergoes a continuous decrease. This phenomenon results in the partial loss of an object’s local structural information and overall contextual relationships, leading to significant semantic differences between shallow and deep features. Specifically, shallow features have been shown to retain more detail and positional information, exhibiting strong equivariant properties that aid in precise object localisation. In contrast, deep features exhibit a propensity to encompass abstract semantic information, characterised by high levels of invariance that facilitate semantic classification. The classification and localisation capabilities of detection models are collectively underpinned by these complementary features.

In the YOLOv5 network, an FPN + PAN architecture is employed to fuse multi-scale features. The Feature Pyramid Network (FPN) primarily conveys high-level semantic information through a top–down approach, while the Path Aggregation Network (PAN) further enhances the expressive power of low-level features. However, prior to the execution of feature fusion between the PAN and FPN structures, it is imperative that YOLOv5 reduces the dimensionality of features from disparate levels through multiple 1 × 1 convolutions. This ensures that the channel counts remain consistent, thus enabling the element-wise addition of the features. This fixed channel alignment approach simplifies the structure to a certain extent but also presents certain issues. Channel dimension inconsistencies lead to imbalanced energy distribution. Forced dimensionality reduction has been shown to disrupt the channel expression structure of original features, causing uneven energy distribution across features of different scales. Fusion conflicts arise from semantic distribution discrepancies. Significant differences in semantic levels between shallow and deep features can cause information interference when directly combined, thereby weakening the discriminative power of multi-scale features. The integration method is limited, and information exchange is constrained. Conventional techniques for straightforward addition or concatenation prove inadequate in facilitating adequate interaction between the shallow and deep layers, consequently leading to suboptimal feature utilisation efficiency.

**Figure 6:**
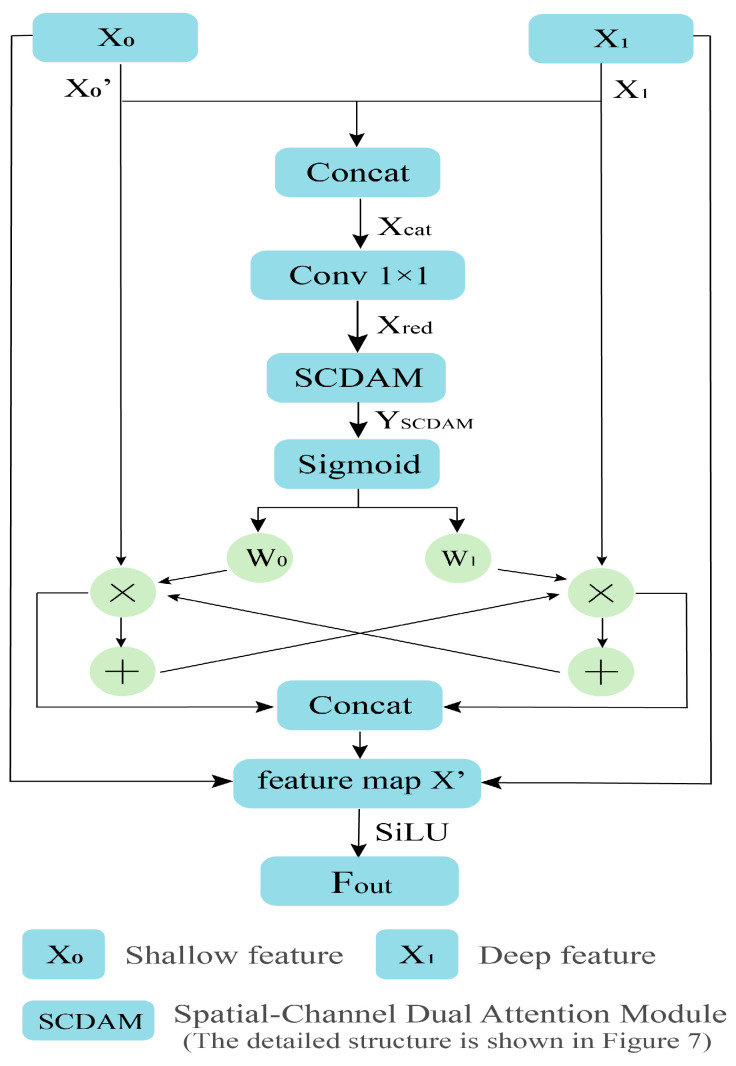
CCFM feature fusion module.

In order to address the aforementioned issues, the present paper presents a Cross Context Fusion Module (CCFM), as illustrated in Figure 6. By introducing a bidirectional cross-fusion mechanism guided by dynamic attention, it effectively resolves the feature deviation issue between shallow-level details and deep-level semantics during multi-scale feature fusion. Structurally, the module achieves channel alignment; in fusion strategy, enables bidirectional information flow; in weight allocation, implements adaptive adjustment; and through a residual architecture, ensures training stability. In comparison with conventional fusion modules, CCFM has been shown to significantly enhance semantic consistency and detail fidelity in feature representation without a substantial increase in parameter count, thereby providing more discriminative features for downstream tasks such as detection and segmentation.

The overall structure of CCFM is illustrated in Figure 6, comprising four principal stages: feature alignment, context concatenation and compression, attention weight generation, and bidirectional fusion with residual enhancement. In the overall architecture (Figure 3), the CCFM module receives two inputs: the upper input, which carries deeper semantic features, is defined as $x_{1}\in R^{B\times C_{1}\times H\times W}$, and the lower input, which carries shallower spatial features, is defined as $x_{0}\in R^{B\times C_{0}\times H\times W}$. Since the features from different levels typically have inconsistent channel numbers, the module first aligns the channels of the shallow features through a 1 × 1 convolution:

(5) $$x_{0}\prime =\left\{\begin{array}{cc} {Conv}_{1\times 1}(x_{0}), & if\;C_{0}=C_{1},\\ x_{0} & \;otherwise. \end{array}\right.$$

where C denotes the number of channels. This operation realigns channels without compromising spatial resolution, thereby establishing the foundation for subsequent feature fusion. Subsequent to channel alignment, shallow and deep features are concatenated along the channel dimension:

(6) $$x_{cat}=concat(x_{0}^{\prime },x_{1})$$

The number of feature channels obtained is twice that of the original. In order to reduce computational complexity and facilitate interaction between information at different levels, CCFM employs a 1 × 1 convolution for compression:

(7) $$x_{red}=Conv_{1\times 1}(x_{cat})$$

Through the above operation, the module ensures sufficient feature fusion while effectively controlling the model’s parameter size and computational overhead. The compressed fused features are then fed into SCDAM, with the objective of generating a dynamic weight map $w\in {\left[0,1\right]}^{B\times C\times 1\times 1}$.

(8) $$w=\sigma (Attention(x_{red}))$$

In the formula, σ(⋅) denotes the sigmoid function. Its role is to map the weights to the [0, 1] range, facilitating the network to adaptively enhance key information.

This weight map dynamically adjusts the response intensity of different channels based on input feature content, thereby achieving adaptive information distribution between shallow and deep features. This enables the network to adaptively select information sources across different semantic levels. Subsequently, employing the generated weights, the fusion outputs for both directions are computed separately:

(9) $$\begin{array}{l} y_{0}=w\cdot x_{0}^{\prime }+(1-w)\cdot x_{1}\\ y_{1}=(1-w)\cdot x_{0}^{\prime }+w\cdot x_{1} \end{array}$$

Shallow features gain partial semantic supplementation from deep features, while deep features also absorb shallow spatial information, enabling bidirectional information flow. Ultimately, the two sets of fusion results are concatenated along the channel dimension, incorporating residual connections and activation functions.

(10) $$F_{out}=SiLU(Concat(\left[y_{0},y_{1}\right],\;\;[x_{0}^{\prime },x_{1}]))$$

where SiLU refers to the Sigmoid Linear Unit activation function. The choice of SiLU over ReLU at this point is mainly based on its smooth and non-monotonic response characteristics, which help to alleviate potential information loss caused by the hard truncation of ReLU during feature fusion.

#### Spatial-Channel Dual Attention Module (SCDAM)

**Figure 7:**
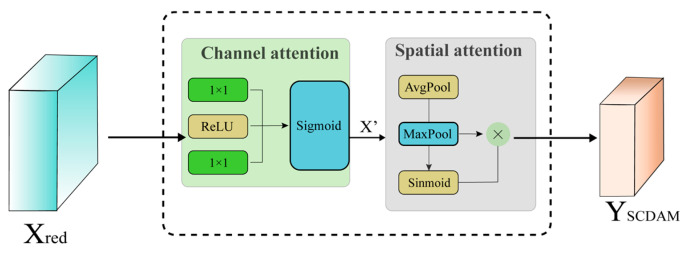
Spatial-Channel Dual Attention Module.

In order to enhance the expressive power of feature maps and increase the network’s focus on critical regions, in this study, we introduced a module called SCDAM. Although the classic Convolutional Block Attention Module (CBAM) [19] also adopts a combined channel and spatial attention design, SCDAM has been optimised in terms of structural design, parameter efficiency, and synergy with CCFM to meet its specific requirements. SCDAM integrates dual attention mechanisms for both channels and spatial dimensions and was developed to enable the adaptive adjustment of the response intensity of feature maps across these dimensions, thereby facilitating more precise feature recalibration. The module is composed of two sub-modules: the channel attention unit and the spatial attention unit. The overall structure of the system is illustrated in Figure 7. The initial step in the process is the weighting of the input features using the channel attention mechanism. This is carried out so that the channel information that is most critical to overall target discrimination can be highlighted. Consequently, the spatial attention mechanism directs the model to concentrate on the regions within the feature map that are most responsive to space.

As shown in Figure 7, the channel attention mechanism is employed to adaptively adjust the importance of different channels across the entire image. Given input features $X_{red}$, they pass through a 1×1 convolutional layer that reduces the number of channels from C to C/r (where r is the reduction parameter used to decrease the computational load). Subsequently, the ReLU activation function enhances the nonlinear expressive capability of the features. The module forms a bottleneck structure through two convolutional layers and the ReLU activation function:

(11) $$CA(X)=\sigma (W_{2}\delta (W_{1}X_{red}))$$

where $W_{1},W_{2}$ represents the weights of the convolutional layer, $\delta \left(\cdot \right)$ denotes the ReLU activation function, $\sigma \left(\cdot \right)$ denotes the Sigmoid function, and r denotes the channel reduction ratio. The generation of the weighted coefficients $CA\in {\left[0,1\right]}^{C}$ for each channel is informed by the modelling of information between channels, resulting in the final output:

(12) $$X^{\prime }=X⊗CA$$

where ⊗ denotes the element-wise multiplication operation at the channel level. This facilitates the suppression of redundant channel features while concomitantly enhancing key semantic channels.

The spatial attention mechanism processes the feature $X^{\prime }$ after channel weighting. The channel-weighted feature $X^{\prime }$ is further input into the spatial attention module to capture the importance of different spatial locations. As shown in the figure, spatial attention extracts spatial saliency information through average pooling and max pooling operations on the channel dimension. Average pooling yields the average response value, while max pooling yields the maximum response value at each location.

(13) $$M_{avg}\left(i,j\right)=\frac{1}{C}\sum_{k=1}^{C}X_{k,i,j}^{\prime }$$

(14) $$M_{\max}\left(i,j\right)=\underset{K\in \left[1,C\right]}{\max}X_{k,i,j}^{\prime }$$

It is evident that both outputs have the shape [B,1,H,W]. Following the concatenation of the data along the channel dimension, the information is fed into a convolutional layer with a kernel size of 7×7. Subsequent to this, a Sigmoid activation is applied, resulting in a spatial weight map.

(15) $$SA\left(X^{\prime }\right)=\sigma \left(f^{7\times 7}\left(\left[M_{avg},M_{\max}\right]\right)\right)$$

The module’s final output is as follows:

(16) $$Y_{SCDAM}=X^{\prime }⊗SA(X^{\prime })$$

Through this operation, the model can focus on salient target regions in the spatial dimension, thereby enhancing the discriminative power and localisation capabilities of features.

## 4. Experimental Results and Analysis

### 4.1. Experimental Environment

The hardware platform and software environment utilised in this experiment are delineated in Table 1.

The YOLOv5 code is version 7.0. The input image resolution for the network is 640 × 640. The batch size has been set to 16, and the training is conducted for 200 epochs. The SGD (stochastic gradient descent) optimiser is utilised, with an initial learning rate of 0.01 and momentum of 0.937. Mosaic data augmentation is employed during the training phase, with all other settings left at their default values.

### 4.2. Experimental Dataset

The experiment utilised a dataset comprising 2912 images of transmission line insulators, with the dataset meticulously annotated using labelling tools such as LabelImg. The dataset was then divided into a training set and a testing set, with the former constituting 80% and the latter comprising the remaining 20%. The training set comprised 2004 images, while the validation set included 908 images. The images encompassed both normal and defective insulator instances, thereby ensuring balanced class distribution. The dataset was divided into three categories: normal insulators, damaged insulators, and flashover insulators and encompassed images captured under a variety of environmental conditions, thereby ensuring that the requisite robustness was achieved.

### 4.3. Evaluation Metrics

mAP is defined as the mean of all category AP values at an IoU threshold of 0.5. The mAP@50:95 is used to denote the mean average precision across IoU thresholds from 0.5 to 0.95. The AP value can be obtained via Formula (17) and also represents the average area under the precision–recall curve. The recall metric is defined as the probability that the model will correctly predict all positive instances. It is important to note that recall is inversely related to the false-negative rate; that is to say, a higher recall indicates a lower false-negative rate. The calculation formula is presented in Equation (18). Precision, also known as the true-positive rate, is defined as the proportion of correctly predicted positives among all predicted positives. The calculation formula is presented in Equation (19).

(17) $$AP=\int_{0}^{1}P\left(r\right)\mathrm{dr}$$

(18) $$P=\frac{TP}{TP+FP}$$

(19) $$R=\frac{TP}{TP+FN}$$

The true positive (TP) is defined as the number of positive samples that have been correctly predicted by the model. The false positive (FP) is the number of negative samples that have been incorrectly predicted as positive. The false negative (FN) is the number of positive samples that have been incorrectly predicted as negative by the model. In this study, a predicted box is considered a TP only if it simultaneously meets the following conditions: (1) IoU ≥ 0.5; (2) the predicted class matches the class of the corresponding ground truth box; (3) the prediction confidence is ≥0.25. If a predicted box has an IoU < 0.5 with all ground truth boxes, or if the IoU meets the threshold but the predicted class is incorrect, it is considered an FP. If a ground truth box is not matched by any predicted box that meets the above criteria, it is considered an FN.

In this paper, we evaluate object detection accuracy using the mAP@0.5 and mAP@0.5:0.95 metrics; FPS is used to assess the number of image frames processed per second. The assessment of model complexity employs two distinct metrics: parameters and GFLOPs.

### 4.4. Analysis of Experimental Results

In this study, we utilised YOLOv5s 7.0 as the baseline model and aimed to enhance its capabilities. Ablation experiments were conducted by sequentially adding the MACF and CCFMs, with the results displayed in Table 2. In Table 3, a performance analysis is presented for different insulator types under identical conditions.

As demonstrated in Table 2, following the integration of the multi-scale attention convolution fusion (MACF) module, the model is capable of extracting feature information within disparate receptive fields through multiple parallel convolutional branches, thereby achieving the simultaneous modelling of local details and global contextual information. This module incorporates an attention mechanism while preserving spatial structural features, thereby enabling the model to focus more on feature responses in key regions. Consequently, it has been demonstrated to enhance feature representation capabilities and information utilisation. Experimental results demonstrate that incorporating the MACF module significantly enhances both the detection accuracy and recall of the model. The mAP@0.5 improves to 0.665, while the mAP@0.5:0.95 ratio increases to 0.432. This improvement validates that our designed three-branch parallel structure effectively fuses features from different receptive fields, thereby alleviating the feature loss problem associated with small insulator defects.

Following the introduction of the Cross-Layer Context-Guided Fusion module (CCFM), the model demonstrates enhanced adaptability during the feature fusion stage. By incorporating a cross-scale information interaction mechanism, the CCFM effectively leverages the complementarity between features at different levels. This enables the model to fully integrate high-level semantic features while preserving spatial details. This module automatically selects feature maps that have been assigned higher scores and that contain richer semantic information for the purpose of object detection. The effect of this process is to improve detection accuracy. A comparison of the baseline YOLOv5 model with the model incorporating CCFM reveals an approximate 2.6% improvement in mAP@0.5, validating the module’s significant role in feature optimisation and information filtering.

The experimental results in Table 3 further demonstrate that different modules significantly improve detection performance across various defect types. For example, for the relatively complex defect types “breakage” and “flashover”, the MC-YOLO model has shown enhanced detection accuracy, reaching 0.600 and 0.449, respectively. This signifies a substantial enhancement in comparison to the original YOLOv5 model. This demonstrates that the enhanced modules effectively strengthen the model’s robustness and generalisation capabilities through multi-scale feature fusion and interaction between pieces of contextual information.

It is evident that the MACF module has a positive impact on the diversity and effectiveness of feature extraction. Furthermore, the CCFM optimises information flow during the feature fusion stage. This facilitates the demonstration of enhanced detection accuracy and stability by the model in complex defect identification tasks. Despite the enhanced model demonstrating a marginal augmentation in parameters and computational intricacy (GFLOPs escalating from 16.0 to 19.0), it maintains commendable real-time performance (FPS ≈ 121), signifying that the proposed structural optimisation achieves a favourable equilibrium between detection capability and computational expenditure.

To validate the effectiveness of the SCDAM attention mechanism, different attention mechanisms were incorporated into the CCFM under identical experimental conditions, and the results are presented in Table 4.

The experimental analysis revealed that the introduction of various attention modules did not result in a significant increase in the model’s parameter count. However, the impact of these modules on the detection accuracy varied considerably. In comparison with the baseline model, attention mechanisms such as SE, ECA, and CA yielded only marginal improvements in accuracy. Although CBAM performs well in general tasks, its performance in this study is inferior to the specially designed SCDAM. The EMA module exhibited marginally superior outcomes, though its precision did not meet expectations. The most significant enhancement in performance was achieved following the integration of the SCDAM attention mechanism. This indicates that SCDAM exhibits superior compatibility with the cross-contextual feature fusion module (CCFM) compared to other attention mechanisms. SCDAM enables more precise weight allocation during feature fusion, thereby effectively enhancing object detection accuracy.

To comprehensively evaluate the overall performance of the proposed MC-YOLO model, we compared it with representative YOLO series models from recent years. Considering the strict requirements for real-time performance and ease of deployment in high-speed railway catenary inspection tasks, this comparative experiment specifically selected lightweight and fast versions from each series, which aligns well with the application scenarios and improvement objectives of our work.

Based on the comparative experimental results shown in Table 5, the proposed model demonstrates significant advantages in balancing accuracy and efficiency. The performance of YOLOv5s, YOLOv8n, YOLOv11, YOLOv12, and MC-YOLO was compared. The results show that YOLOv8n achieved the fastest inference speed at 270 FPS, while YOLOv11 and YOLOv12 exhibited higher accuracy with mAP@0.5 values of 0.667 and 0.651, respectively. In contrast, MC-YOLO achieved the highest mAP@0.5 value of 0.674 among all compared models while maintaining a relatively low parameter count and inference speed, representing a 4.1% improvement over the original YOLOv5s baseline model.

Despite achieving an accuracy of 0.711, YOLOv9c has a parameter count of 25.3 M and an inference speed of only 57 FPS. Models such as YOLOv6s and YOLOv8n, while offering advantages in terms of speed, exhibit detection accuracies that are lower than those of the proposed method. The experimental results demonstrate that the proposed model achieves superior detection accuracy while maintaining a moderate parameter size and high inference efficiency, reflecting excellent overall performance.

### 4.5. Visual Analysis

**Figure 8:**
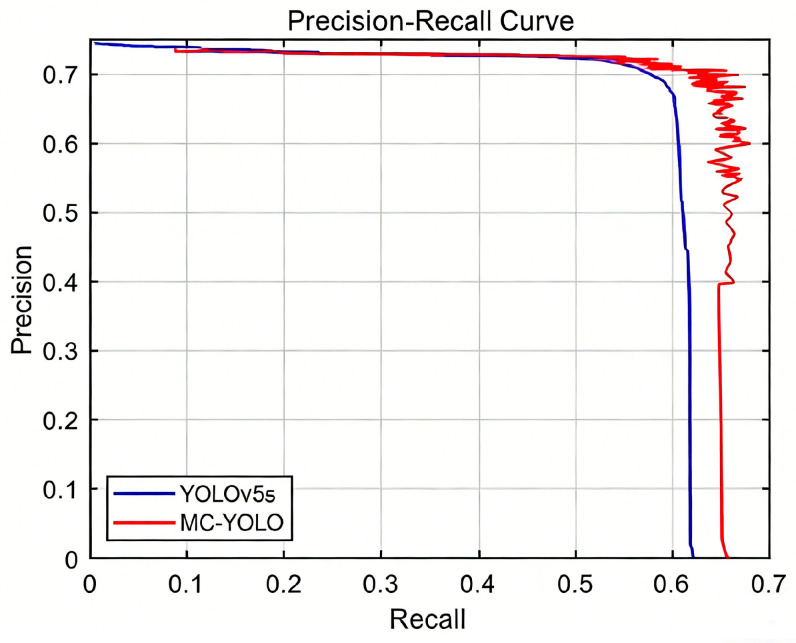
Comparison chart of mAP before and after improvement.

As demonstrated in Figure 8, the MC-YOLOmAP@0.5 model exhibited a 3.1% enhancement over the baseline model. The proposed enhancement method has been demonstrated to be effective in optimising the performance of the MC-YOLO model curve, which integrates all enhancement strategies. The result is the maximum coverage area in terms of both the horizontal and vertical axes. This finding suggests that MC-YOLO achieves the highest levels of precision and recall across all detection categories, and that its enhancements effectively enhance the detection capability for insulators on railway overhead contact systems.

**Figure 9:**
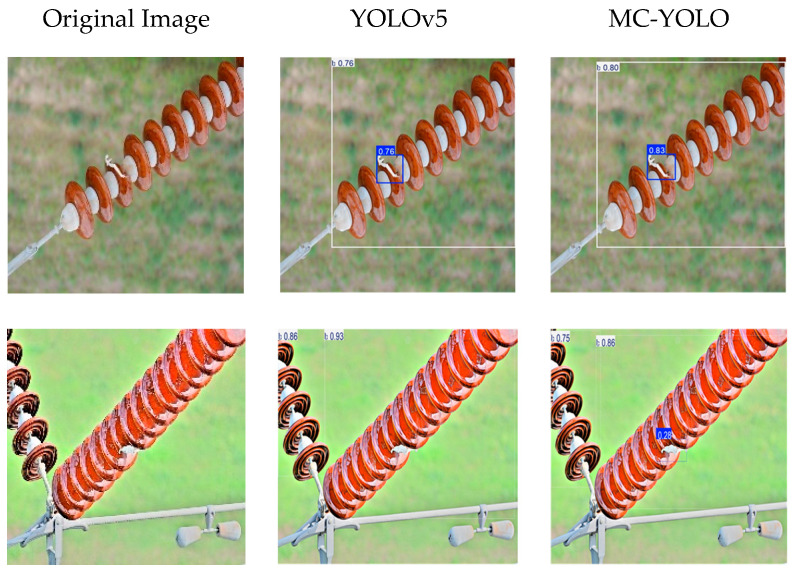

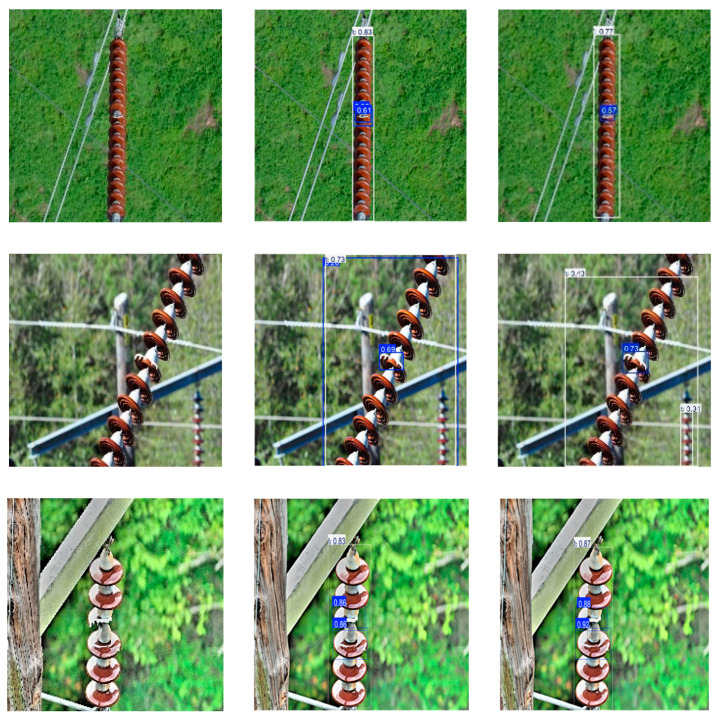
Detection performance of YOLOv5 and MC-YOLO.

In order to provide a more intuitive demonstration of the detection performance between MC-YOLO and YOLOv5, images were randomly selected from the dataset for detection, as illustrated in Figure 9.

As demonstrated in Figure 9, under real-world conditions and in the presence of natural lighting, the MC-YOLO model exhibits a substantially higher level of detection performance in comparison to YOLOv5. Specifically, MC-YOLO demonstrates notable robustness in densely populated target areas, with its predicted bounding boxes generally achieving higher confidence scores. For targets that are densely clustered, YOLOv5 exhibits significant false-negative and false-positive results, whereas MC-YOLO achieves complete and accurate detection. In scenarios involving minute objects, YOLOv5 demonstrates deficiencies in its perception capabilities, while MC-YOLO exhibits a capacity for precise recognition. Furthermore, YOLOv5 is susceptible to false detections against complex backgrounds, such as misclassifying damaged structures. The proposed MC-YOLO algorithm has been demonstrated to achieve an optimal balance of accuracy and speed in the detection of defects in insulators. This has resulted in a significant reduction in both false-negative and false-positive rates, ensuring greater alignment with practical detection requirements.

**Figure 10:**
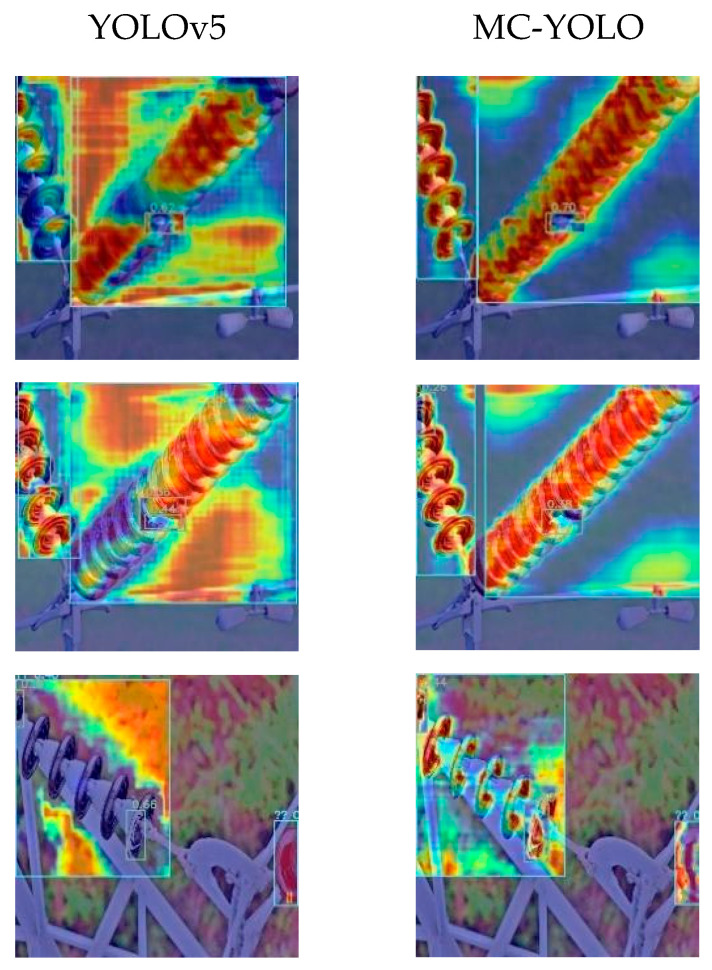
Grad-CAM visualisation.

In conclusion, in order to provide a more precise evaluation of the model’s detection capabilities, a visualisation analysis was conducted utilising the Gradient-weighted Class Activation Mapping (Grad-CAM) technique. Grad-CAM is a visualisation technique that highlights key image regions through heatmaps to visualise model decisions, clearly displaying the location and area of the target that the model is attempting to predict within the image [25]. Furthermore, Grad-CAM enables a better understanding of the model during object detection and aids in evaluating its detection capabilities, as shown in Figure 10.

Figure 10 presents a visual comparison between the YOLOv5 model and the MC-YOLO model. As demonstrated in the category activation maps, MC-YOLO exhibits stronger responses, more precise localisation, and superior resistance to background interference. In the figure, warm colors like red and yellow show important areas, while cool colors like blue show less important areas. This finding suggests that MC-YOLO generates more accurate predictions and has superior recognition capabilities, enhancing its application prospects across a range of domains.

## 5. Conclusions

The main object detection algorithms employed in the field of insulator detection have demonstrated a lack of precision and have thus fallen short of the requisite standards for practical applications. In order to enhance precision in detecting railway catenary insulators whilst minimising the complexity of the model, an algorithm has been proposed. It is known as MC-YOLO and is designed to improve accuracy whilst maintaining real-time performance. The modules were designed to effectively extract multi-scale information and enhance the model’s ability to represent feature information. Subsequently, a cross-context feature fusion module (CCFM) was developed. It has been shown that shallow features benefit from partial semantic supplementation by deep features while deep features absorb shallow spatial information, thereby facilitating bidirectional information flow. The Spatial-Channel and Attention Module (SCDAM) was integrated into CCFM. The integration of a dynamic attention-guided bidirectional cross-fusion mechanism has been shown to effectively address the discrepancy between shallow details and deep semantics during multi-scale feature fusion. The MC-YOLO algorithm achieved an mAP0.5 of 67.4% on the dataset presented in this paper, with a frame rate of 121 FPS, enabling the rapid and accurate detection of insulator defects. To promote the transition of this research from laboratory validation to practical engineering applications, we further envision its industrial implementation pathway. Specifically, MC-YOLO can be embedded as the core detection engine within drones or intelligent inspection platforms. This enables real-time detection and preliminary alerts on edge devices, while complex sample verification and continuous model optimisation are performed in the cloud, thereby forming a comprehensive intelligent inspection system. To address practical challenges in field inspections—such as variable lighting and complex weather conditions—enhancing the model’s environmental robustness requires constructing incremental datasets and incorporating domain adaptation techniques [26]. Furthermore, the small target and complex background detection problems addressed by this method are broadly applicable, and its application scenarios can naturally extend to fields such as high-voltage power line inspection, substation equipment monitoring, and surface defect detection of infrastructure, including wind turbine blades and bridge cables [27,28,29]. Notably, the current model has increased parameter volume due to the introduction of attention mechanisms. Future work will employ lightweight techniques like model pruning and quantisation compression to further optimise computational efficiency while maintaining accuracy, thereby meeting practical deployment requirements for embedded devices [30,31,32]. Through continuous refinement along this technical trajectory, MC-YOLO holds promise to evolve into a stable and reliable automated inspection tool, delivering a practical technical solution for intelligent operation and maintenance of power systems.

## Figures and Tables

**Table 1:** Experimental configuration information.

Name	Parameters
GPU	RTX 3060
CPU	6 x E5-2680 v4
Operating System	Linux ubuntu22.04
CUDA	11.1
Programming Language	Python 3.10
Pytorch	2.0.1

**Table 2:** Ablation experiments (√ indicates that the corresponding module in the column is enabled).

MACF	CCFM	Precision	Recall	Parameters	GFLOPs	mAP@0.5	mAP@50:95	FPS
		0.719	0.622	7,018,216	16.0	0.633	0.411	169
√		0.711	0.671	7,804,776	17.6	0.665	0.432	125
√	√	0.722	0.660	8,215,536	19.0	0.674	0.436	121

**Table 3:** Ablation experiments for a single module.

Model	Defect	GFLOPs	Params	Precision	Recall	mAP@0.5	mAP@50:95
YOLOv5	Normal	16.0	7,018,216	0.97	0.954	0.975	0.745
	Damage	16.0	7,018,216	0.645	0.552	0.558	0.275
	Flashover	16.0	7,018,216	0.543	0.389	0.365	0.223
YOLOv5 + MACF	Normal	17.6	7,804,776	0.953	0.95	0.978	0.734
	Damage	17.6	7,804,776	0.660	0.587	0.595	0.299
	Flashover	17.6	7,804,776	0.519	0.475	0.423	0.243
MC-YOLO	Normal	19.0	8,215,536	0.963	0.956	0.980	0.746
	Damage	19.0	8,215,536	0.662	0.571	0.600	0.302
	Flashover	19.0	8,215,536	0.542	0.456	0.449	0.260

**Table 4:** Attention comparison experiments.

Model	Params (M)	GFLOPs	mAP@0.5	mAP@0.5:0.95
YOLOv5	7.02	16.0	0.633	0.411
SCDAM	8.19	19.0	0.674	0.436
EMA [20]	8.17	19.4	0.671	0.435
SE [21]	8.16	18.8	0.668	0.432
CBMA [22]	8.16	18.8	0.659	0.427
CA [23]	8.16	19.0	0.656	0.430

**Table 5:** Contrast experiment.

Model	Params (M)	mAP@0.5	FPS
YOLOv5s	7.1	0.633	169
YOLOv6s [24]	4.2	0.597	276
YOLOv7	37.4	0.551	50
YOLOv7-tiny	6.1	0.476	126
YOLOv8n	3.2	0.664	270
YOLOv9c	25.3	0.711	57
YOLOv10	8.1	0.617	65
YOLOv11	9.4	0.667	182
YOLOv12	9.2	0.651	133
Ours	8.2	0.674	121

## Data Availability

The original contributions presented in the study are included in the article; further inquiries can be directed to the corresponding author.
